# Facile Synthesis of FeS@C Particles Toward High-Performance Anodes for Lithium-Ion Batteries

**DOI:** 10.3390/nano9101467

**Published:** 2019-10-16

**Authors:** Xuanni Lin, Zhuoyi Yang, Anru Guo, Dong Liu

**Affiliations:** 1 College of Chemical Engineering, Beijing University of Chemical Technology, Beijing 100029, China; Elainelxn@126.com (X.L.); yegbert@163.com (Z.Y.); 2 Aerospace Research Institute of Materials & Processing Technology, Beijing 100076, China

**Keywords:** scalable synthesis, nanoconfined FeS, porous carbon, iron(III) p-toluenesulfonate, lithium-ion batteries

## Abstract

High energy density batteries with high performance are significantly important for intelligent electrical vehicular systems. Iron sulfurs are recognized as one of the most promising anodes for high energy density lithium-ion batteries because of their high theoretical specific capacity and relatively stable electrochemical performance. However, their large-scale commercialized application for lithium-ion batteries are plagued by high-cost and complicated preparation methods. Here, we report a simple and cost-effective method for the scalable synthesis of nanoconfined FeS in porous carbon (defined as FeS@C) as anodes by direct pyrolysis of an iron(III) p-toluenesulfonate precursor. The carbon architecture embedded with FeS nanoparticles provides a rapid electron transport property, and its hierarchical porous structure effectively enhances the ion transport rate, thereby leading to a good electrochemical performance. The resultant FeS@C anodes exhibit high reversible capacity and long cycle life up to 500 cycles at high current density. This work provides a simple strategy for the mass production of FeS@C particles, which represents a critical step forward toward practical applications of iron sulfurs anodes.

## 1. Introduction

Lithium-ion batteries (LIBs) have gained great success as an energy storage technology for portable electronics due to their stable electrochemical performance, low cost, high energy density, and environmental compatibility [1,2,3]. However, next generation LIBs with greater energy density, calendar life, and safety need to be developed for large-scale energy storage applications such as electric vehicles, smart power grids, internet of things (IoT), etc. [4]. With ever-increasing energy storage requirements, developing new electrode materials with a high specific capacity and stable electrochemical performance has emerged as a very promising solution to increase the energy density and cycle life of LIBs [4]. For example, electrode materials with high specific capacity have been extensively researched for LIBs, including Si, [5,6,7] Sn, [8,9] metal oxides, and metal sulfides (MSs) [10,11], etc. As high specific capacity anodes suffer from volume expansion, organic polymeric binders show significant improvement on their electrochemical performance [12]. Specially, self-healing polymers can repair damage spontaneously, leading to the stable operation of high-capacity anodes [13]. Substantial progress has been made toward resolving the issues caused by volume change during the charge/discharge process, although there are still some problems. Among these high specific capacity anodes, metal sulfides have been attracting much attention owing to their good rate capacity and cycling performance [10]. Therefore, developing metal sulfides as anodes for LIBs is significantly important.

Metal sulfides have been extensively applied in versatile fields, such as energy transformation and storage [14,15,16,17,18] and photonic/electronic devices and sensors, [19,20,21], mainly due to their tunable stoichiometric compositions, unique crystal structure, quantum size effects, and optical/electrical properties, etc. [20,22,23]. As anode materials, MSs are promising candidates for LIBs owing to their high specific capacity and relatively low cost [14,24,25]. Compared to silicon and metal oxides, metal sulfides possess a better rate capacity and cycling performance due to their relatively lower volumetric expansion/shrinkage during the lithiation/delithiation process [26]. Among all reported MSs, iron sulfides have witnessed increasing concerns owing to their low cost, earth-abundance, and high theoretical capacity [27]. However, electrochemical performances of the iron sulfide anodes for LIBs are plagued by the possible dissolution of sulfur, poor electrical conductivity, and volume expansion caused by the multiple electron transfer per iron ion through the conversion reaction [28,29]. According to an earlier report, iron sulfide/C hybrid engineering is promising in addressing the disadvantages of iron sulfide anodes because a carbon matrix not only increases the electron transport rate but also buffers large volume expansion/shrinkage [28]. In addition, the carbon coating wrapped onto the sulfides can reduce polysulfide dissolution into the electrolyte [29]. Several approaches have been developed for preparing iron sulfide/C hybrid anodes, including solution reaction [30], electrospinning [31], the template method [29,32], gas-phase reaction [33], etc. Some interesting macromolecular architectures are expected to use as porous carbon precursors for constructing iron sulfide/C hybrid anodes [34,35,36]. Although significant progress has been achieved for constructing iron sulfide/C hybrid anodes, the majority of approaches are still complicated for mass production of the iron sulfides/C hybrid anodes with a unique structure and stable electrochemical performance. For example, these methods have to prepare complex precursors or use expensive graphene. Therefore, developing a simple, reliable, and cost-effective method for preparing iron sulfide/C particles is significantly important and urgent.

Here, we report a simple and cost-effective method for the scalable fabrication of iron sulfide/C hybrid anodes by nanoconfining FeS in porous carbon directly by the one-pot pyrolysis of iron(III) p-toluenesulfonate hexahydrate (denoted as IPTH) under Ar atmosphere. The IPTH precursor contains abundant sulfur, carbon, and iron elements. Thermal decomposition of IPTH could not only obtain FeS nanoparticles but also formed a carbon conductive network, leading to nanoconfined FeS in porous carbon. The resultant FeS@C particles exhibited a high reversible specific capacity and long-term cycle stability for LIBs. This study on the newly developed approach for FeS@C particles provides a scalable production method for high-performance anodes, which may open up new avenues for the commercialized development of LIBs with high energy density and a long cycle life.

## 2. Materials and Methods

### 2.1. Preparation of Materials

The FeS@C particles were prepared by direct pyrolysis of iron(III) p-toluenesulfonate at a setting temperature with a ramp rate of 5 °C min^−1^ and kept at this temperature for 2 h under Ar atmosphere. The pyrolysis temperature was set to 600 °C, 700 °C, and 800 °C, producing FeS@C-600, FeS@C-700, and FeS@C-800 particles, respectively.

### 2.2. Material Characterization

X-ray powder diffraction (XRD) patterns were performed by a Bruker D2 PHSDER X-ray (Bruker AXS, Portland, OR, USA) diffractometer with filtered Cu Kα radiation (λ = 1.54056 Å). Raman spectra were measured using a Raman spectrometer (Horibra LabRAM HR Evolution, Kyoto, Japan) with a laser of 514 nm. X-ray photoelectron (XPS) analysis was tested by an ESCALAB MKII X-ray photoelectron spectrometer (VG Instruments, San Francisco, CA, USA) with the Mg Kα x-ray radiation under a pressure of 2 × 10^−9^ Torr. Thermogravimetric analysis (TGA) was carried out with a Mettler Toledo TGA/DSC 3+ (Mettler-Toledo, Millville, Mass, USA) at a scanning rate of 10 °C min^−1^ under Ar or O_2_ atmosphere. The surface area was measured using the Brunauer–Emmett–Teller (BET) (Micromeritics, Norcross, GA, USA) method (ASAP 2460 N_2_ adsorption apparatus) at 77.3 K; pore-size distribution curves were obtained from N_2_ adsorption data. Scanning electron microscope (SEM) images were obtained on a S-4700 field emission scanning electron microscope (Hitachi, Tokyo, Japan). Transmission electron microscope (TEM) and high-resolution TEM (HRTEM) images were obtained by using a JEM 2100 (JEOL, Tokyo, Japan) TEM operated at an accelerating voltage of 200 kV. The elemental mappings were collected on a STEM unit with a high-angle, annular dark field (HAADF) detector (FEI Tecnai G2 F20, 200 kV, Hillsboro, OR, USA).

### 2.3. Electrode Preparation and Electrochemical Measurement

The electrochemical performances were tested by galvanostatic cycling of coin cells with a FeS@C electrode as working electrode and lithium foil as a counter electrode. All electrodes were prepared using the casting method with the active materials (80 wt%), Super P carbon black (10 wt%), and polyvinylidene fluoride (PVDF) (10 wt%). The electrolyte for all tests consisted of 1.2 M LiPF_6_ in ethylene carbonate (EC) and diethylene carbonate (DEC) (1:1 w/w). All coin cells were assembled in an Ar-filled glovebox. The electrochemical measurements were conducted by galvanostatic cycling of 2032-type coin half-cells using Li foil (99.9%, China Energy Lithium Co. Ltd., Tianjin, China) as the counter electrodes. Galvanostatic charge/discharge measurements were conducted over a voltage range of 0.01–3 V at room temperature by a Land battery test system (Land, Wuhan, China). The cyclic voltammetry (CV) measurements were carried out using an electrochemical workstation (Solar tron SI 1260, AMETEK, Hampshire, UK) at a scan rate of 0.2 mV s^−1^ in the voltage range of 0.01–3 V (vs. Li/Li^+^). The electrochemical impedance spectroscopy (EIS) experiments were performed on an electrochemical workstation (Autolab, Metrohm, Herisau, Switzerland) in the frequency range of 10 Hz to 0.1 Hz.

## 3. Results and Discussion

### 3.1. Morphology and Structure

**Figure 1:**
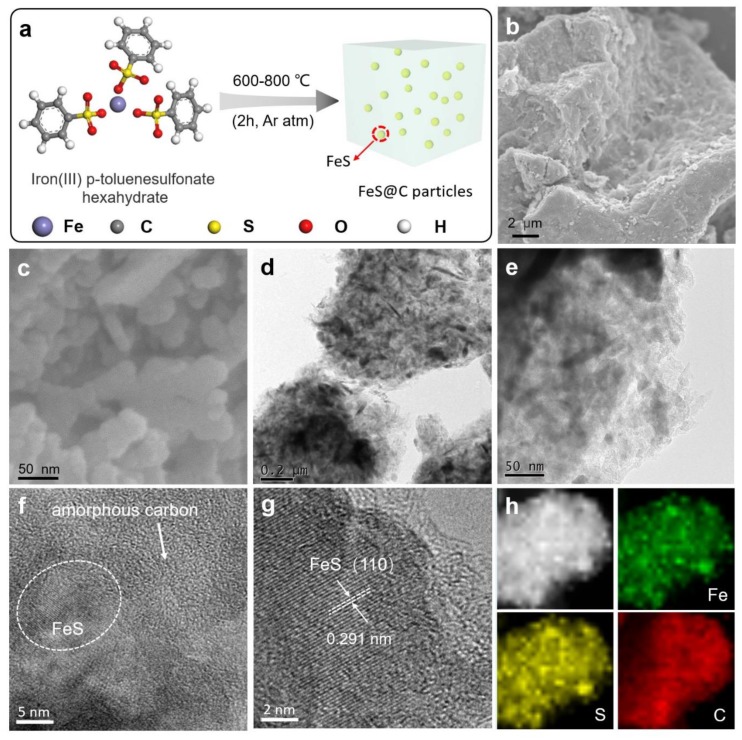
(**a**) Schematic illustration of the preparation process for the FeS@C particles. Iron(Ⅲ) p-toluenesulfonate hexahydrate was directly calcined at the desired temperatures under Ar atmosphere, obtaining FeS@C particles. The FeS nanoparticles were nanoconfined into a carbon matrix. (**b**,**c**) SEM images of the FeS@C particles. (**d**,**e**) TEM images of the FeS@C particles; (**f**,**g**) high-resolution TEM (HRTEM) images of the FeS@C particles. (**h**) corresponding high-angle annular dark field scanning transmission electron microscopy (HAADF-STEM) and element mapping images of the FeS@C particles.

The synthetic procedure for FeS@C particles is depicted in Figure 1a. The iron(III) p-toluenesulfonate hexahydrate precursor containing iron, sulfur, carbon, and oxygen elements was heated up to 600–800 °C with a ramp rate of 5 °C min^−1^ and kept for 2 h under Ar atmosphere, producing FeS@C-600, FeS@C-700, and FeS@C-800 particles, respectively. During the pyrolysis process, iron and sulfur elements of the IPTH precursor formed FeS nanoparticles, and benzenesulfonic acid transformed to a carbon network, leading to a one-step formation of the FeS@C particles by nanoconfining FeS nanoparticles in porous carbon materials. The production of FeS@C particles can be scaled up with low-cost via one-pot pyrolysis of IPTH, as shown in Figure S1 (Supporting Information). The yield of the FeS@C particles was calculated to be about 52% at 700 °C by the weighing method, which can be further identified by thermogravimetric (TG) data (Figure S2, Supporting Information). TG and the corresponding differential thermogravimetry (DTG) curves of the IPTH precursor under Ar atmosphere (Figure S2, Supporting Information) showed that there were three main mass losses. The first and second mass losses in the range of 30–448 °C were about 15.11% by mass and corresponded to the elimination of crystal water, which was close to the theoretical water content of the IPTH (15.94%). The third mass loss occurred from 448 °C and indicated that iron(III) p-toluenesulfonate decomposed and converted most of the thermally stable benzene domain into graphitic carbon embedded with FeS nanoparticles. After high temperature treatment of FeS@C particles in O_2_ atmosphere, the C and FeS transformed into CO_2_ and Fe_2_O_3_ [32]. The mass ratio of FeS to C in the FeS@C samples can be calculated by the residual Fe_2_O_3_ from the TGA curves. As shown in Figure S9, the mass ratio of FeS to C for the FeS@C-600, FeS@C-700, and FeS@C-800 can be calculated to 0.395, 0.69, and 0.43, respectively.

**Figure 2:**
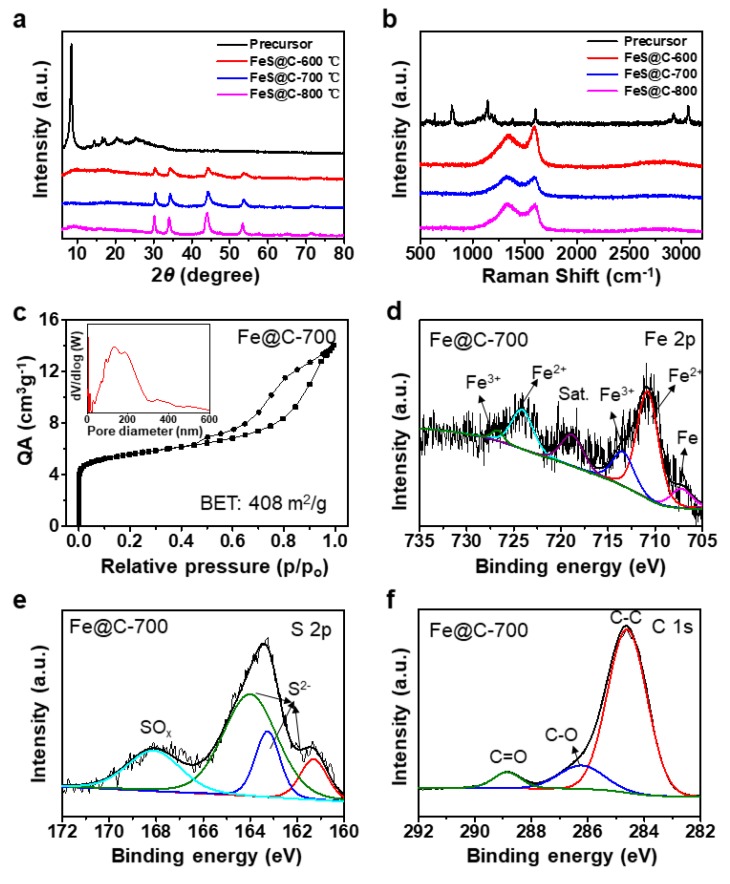
(**a**) The XRD patterns and (**b**) Raman spectra of the precursor, FeS@C-600, FeS@C-700, and FeS@C-800 particles. (**c**) Nitrogen adsorption/desorption isotherms and corresponding pore-size distribution (inset in Figure 2c) for the FeS@C-700 particles. (**d**–**f**) High-resolution X-ray photoelectron spectroscopy (XPS) spectra of Fe 2p, S 2p, and C1s for the FeS@C-700 particles.

Figure S3 (Supporting Information) shows digital photo images of the IPTH precursor before and after pyrolysis at 700 °C, from which it can be observed that the original yellow IPTH precursor was transformed to black FeS@C particles. Scanning electron microscopy (SEM) images of the FeS@C particles given in Figure 1b–c clearly show that the bulk particles were composed of a lot of small particles with a diameter of 15–50 nm. The unique structure of the FeS@C particles can be clearly observed in Figure S4 (Supporting Information). Transmission electron microscopy (TEM) images in Figure 1d–e reveal that the small FeS nanoparticles were embedded into an amorphous carbon matrix, which was vital for rapid electrical transport for the FeS nanoparticles. Enlarged view of the TEM image in Figure 1f revealed that the FeS nanoparticles were nanoconfined in amorphous carbon, suggesting the formation of the FeS@C composite particles. High resolution TEM (HR-TEM) image given in Figure 1g showed FeS nanoparticles with lattice fringes of 0.291 nm, corresponding to the (110) crystal planes and crystalline property of the FeS nanoparticles [29]. Scanning transmission electron microscope (STEM) image and element mapping of the FeS@C particles given in Figure 1h exhibited that a uniform distribution of Fe, S and C elements were observed, indicating FeS nanoparticles were homogeneously embedded into a carbon matrix. SEM and TEM images given in Figure 2 clearly showed that the FeS nanoparticles were nanoconfined into a carbon matrix, which can not only boost electron transport but also buffer large volume expansion/shrinkage of FeS particles, leading to a high electrochemical performance for lithium-ion batteries.

X-ray diffraction (XRD) patterns of the IPTH precursor before and after thermal treatment were recorded. As expected, XRD patterns of all three annealed samples at 600 °C, 700 °C, and 800 °C in Figure 2a showed the respective characteristic peaks of FeS, where peaks located at 30.3°, 34.3°, 44.3°, and 53.7° are ascribed to (110), (112), (114), and (300) planes of FeS (troilite-2H) (JCPDS # 37-0477) [29,37,38]. No peaks below 700 cm^−1^ can be observed from the Raman spectra of FeS@C-700 particles in Figure S5 (Supporting Information), further proving that the iron sulfide in FeS@C particles possessed a troilite phase [38]. Raman spectra of the IPTH precursor before and after annealing given in Figure 2b clearly exhibit the D- and G-bands at 1337 and 1588 cm^−1^, suggesting that the IPTH precursor transformed into amorphous carbon. The G-band at 1588 cm^−1^ is associated with the E2g mode, while the D-band located at 1337 cm^−1^ corresponds to the defect mode [39]. The high graphitization degree of these three samples is evident from the high ratio of G-band/D-band (over 1), leading to an improved electrical conductivity. The XRD pattern (Figure 2a) and Raman spectra (Figure 2b) confirm that thermal treatment converted the IPTH precursor into FeS@C particles with good electrical conductivity, ensuring rapid electron transport for LIBs.

Nitrogen adsorption–desorption isotherms were performed at 77 K to research the porosity of the FeS@C particles. As shown in Figure 2c, a sharp gas adsorption was found at low pressure (P/P_0_ < 0.1) for the FeS@C-700 particles, which revealed the presence of micropores. The rapid nitrogen uptake (P/P_0_ > 0.9) might be due to the presence of secondary, much larger pores. The type IV isotherm curves (Figure 2c) show an obvious hysteresis and further confirm the existence of mesopores. Barrett–Joyner–Halenda (BJH) pore-size distribution curves derived from N_2_ desorption (inset in Figure 2c) confirm the presence of micropores, mesopores, and macropores with diameters from 2 nm to 300 nm and a pore volume of 0.36 cm^3^ g^−1^. The nitrogen adsorption–desorption isotherms and BJH pore-size distribution curves of the FeS@C-600 and FeS@C-800 particles were similar to that of FeS@C-700 particles, as shown in Figure S6 (Supporting Information). The Brunauer–Emmett–Teller (BET) surface areas were 383 m^2^ g^−1^ for the FeS@C-600 particles, 408 m^2^ g^−1^ for the FeS@C-700 particles, and 416 m^2^ g^−1^ for the FeS@C-800 particles (Figure 2c and Figure S6, Supporting Information) and were slightly enlarged on increasing pyrolysis temperature. Overall, the above data confirm that the FeS@C particles possessed a large surface area, high pore volume, and wide pore-size distribution. The hierarchical porous structure of the FeS@C particles is beneficial for rapid ion transport, leading to a high rate performance [40].

Typical X-ray photoelectron spectroscopy (XPS) for three FeS@C particles provided in Figure S7 clearly shows typical peaks for the iron, sulfur, and carbon elements of the FeS@C particles, which originated from the IPTH precursor. As shown in Figure 2d, the fitted XPS peaks for Fe 2p of the FeS@C-700 particles centered at 710.8 eV and 724.1 eV were attributable to characteristic peaks of Fe^2+^ [41,42]. The high-resolution XPS (HR-XPS) S 2p spectrum (Figure 2e) can be deconvoluted into four different peaks. Peaks at 161.3, 163.26, and 164 eV correspond to the typical peaks of S^2-^ [41,43], and the peak at 168.1 eV corresponds to SO_3_^2-^ [44]. Peaks at 284.6, 286.2, and 288.85 eV observed in the HR-XPS C 1s spectrum (Figure 2f) can be assigned to “C–C/C=C”, “C–O”, and “C=O” bonds, respectively [39,45]. The above results in Figure 2d–f suggest the successful preparation of the FeS@C-700 particles. The HR-XPS Fe 2p, S 2p, and C 1s spectra in Figure S8 (Supporting Information) confirm that the FeS@C-600 and FeS@C-800 particles were also successfully prepared.

### 3.2. Battery Performance

**Figure 3:**
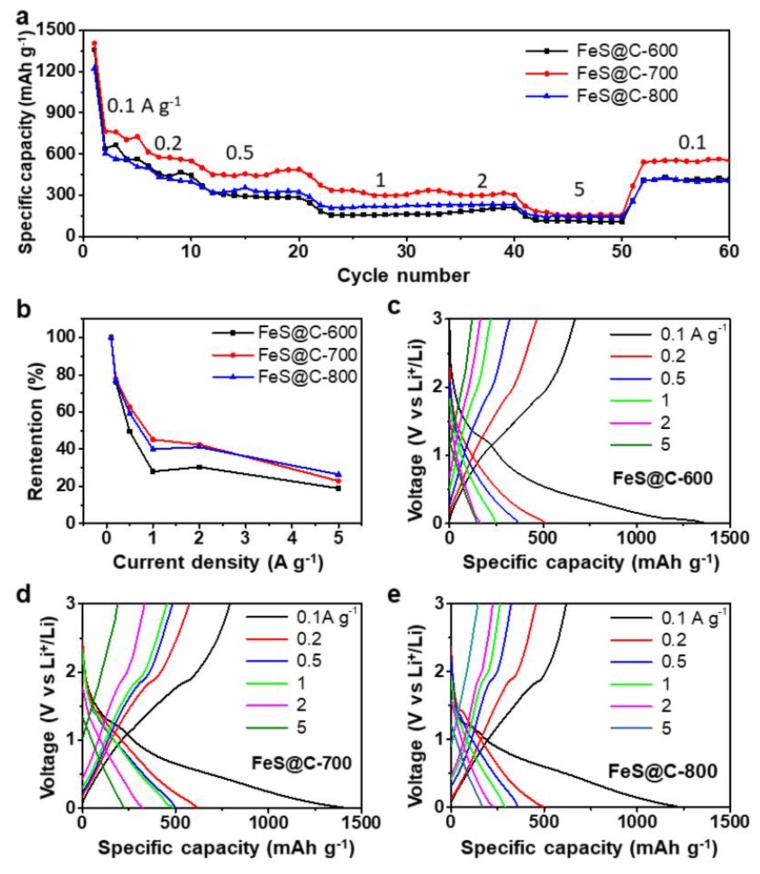
(**a**) Rate capability of the FeS@C electrodes at various current densities from 100 mA g^−1^ to 5000 mA g^−1^. The mass loading of three electrodes is over 1.0 mg cm^−2^. (**b**) Retention of specific capacity as a function of various current densities from 100 mA g^−1^ to 5000 mA g^−1^ for three different electrodes. (**c**–**e**) Charge/discharge profiles of FeS@C-600, FeS@C-700, and FeS@C-800 electrodes at various current densities.

Figure 3a shows the rate performance of the FeS@C-600, FeS@C-700, and FeS@C-800 electrodes. As shown in Figure 3b, the FeS@C-700 and FeS@C-800 electrodes displayed better a rate capability than the FeS@C-600 electrode (Figure 3b), which can be explained by enhancing the electrical conductivity of FeS@C particles with an increasing annealing temperature. The electrical conductivity was 0.05 S m^−1^ for FeS@C-600 particles, 5.18 S m^−1^ for FeS@C-700 particles, and 7.23 S m^−1^ for FeS@C-800 particles, measured by using four-point probe resistance technology. A high electrical conductivity of electrode materials can boost the rapid electrical transport, leading to high rate performance [39]. The deduction can be further proved by electrochemical impedance spectra (EIS) of the FeS@C-600, FeS@C-700, and FeS@C-800 electrode measured before cycling, as shown in Figure S9 (Supporting Information). The semicircle in the Nyquist plots reflects charge-transfer resistance in the electrode. The initial resistance of the FeS@C-700 (107 Ω) and FeS@C-800 (85 Ω) electrodes is smaller than that of the FeS@C-600 electrode (209 Ω). The FeS@C-700 electrode exhibited high reversible-average-specific capacities of 739, 575, 464, 336, 314, and 158 mAh g^−1^ at current densities of 100, 200, 500, 1000, 2000, and 5000 mA g^−1^, respectively. After several cycles with increasing current density, the specific capacity of the electrode can be returned to 532 mAh g^−1^ at a current density of 100 mA g^−1^. The FeS@C-700 electrode delivered a much higher specific capacity at different current densities than the FeS@C-800 electrode because the FeS@C-700 particles possessed a high initial specific capacity owing to the high content of FeS in the FeS@C particles (Figure S10, Supporting Information). These results show that the FeS@C electrodes possess a superior rate performance.

**Figure 4:**
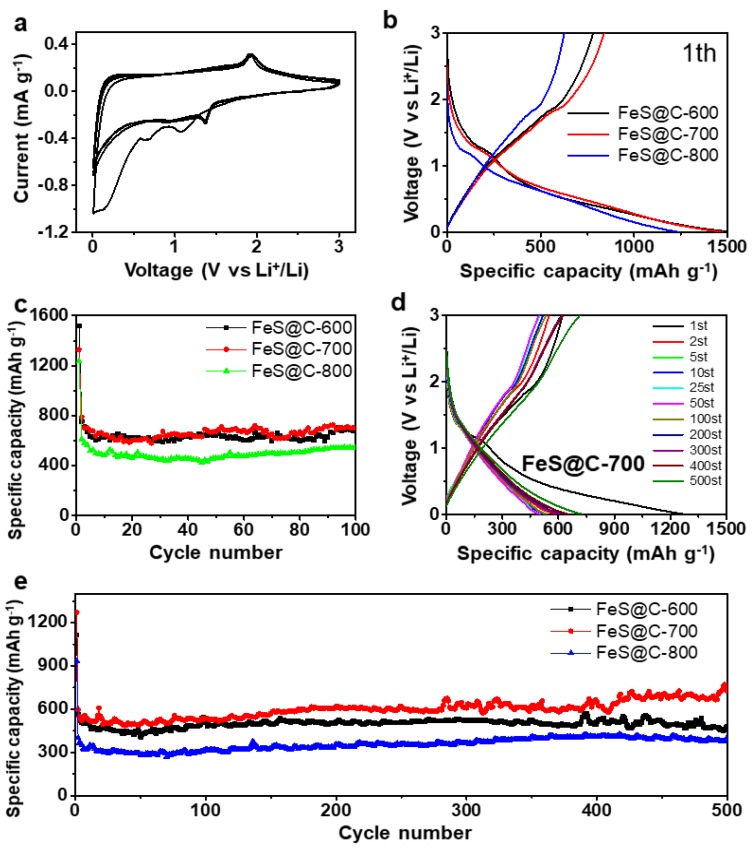
(**a**) Representative cyclic voltammograms of FeS@C-700 electrodes at a scan rate of 0.02 mV s^−1^. (**b**) First charge/discharge profiles of FeS@C-600, FeS@C-700, and FeS@C-800 electrodes at a current density of 100 mA g^−1^. (**c**) Cycle performance of FeS@C-600, FeS@C-700, and FeS@C-800 electrodes at a current density of 100 mA g^−1^. (**d**) Charge/discharge profiles of the FeS@C-700 electrode at a current density of 500 mA g^−1^. (**e**) Long cycling stability of FeS@C-600, FeS@C-700, and FeS@C-800 electrodes at a current density of 500 mA g^−1^. The mass loading of the three electrodes is over 1.0 mg cm^−2^.

The electrochemical behavior of the FeS@C-600, FeS@C-700, and FeS@C-800 electrodes was researched. The representative discharge–charge profiles of these three electrodes at current densities of 100, 200, 500, 1000, 2000, and 5000 mA g^−1^ are shown in Figure 3c–e, respectively. The distinct discharge–charge plateaus are centered at around 1.3 V, corresponding to the lithiation of FeS [33]. The FeS@C-700 and FeS@C-800 electrodes exhibited more lithium storage/release than the FeS@C-600 electrode (Figure 3b). The electrochemical behavior of the FeS@C electrode was further analyzed by using cyclic voltammograms (CVs) in the voltage range of 0.01–3 V at a sweep rate of 0.2 mV s^−1^. Figure 4a presents the first five CV curves of the FeS@C-700 electrode; the peak at 1.08 V in the first cathodic scan is ascribed to the formation of Fe (FeS + 2Li^+^ + 2e^−^ = Li_2_S + Fe) [27,29,46]. A broad peak appearing from 0.75 V to 0 V corresponds to the formation of a solid–electrolyte-interphase (SEI) layer on the surface of the FeS@C particles [29,33], which was confirmed by the peak disappearing in the later cathodic scan. Peaks at 1.91 V in the first scan correspond to the oxidation reaction of Fe with Li_2_S, forming FeS [29,46]. The reduction peaks shifted to 1.37 V in the discharge cycles (2nd–5th), while the oxidation peaks at 1.94 V in the charge cycles (2nd–5th) did not show an obvious shift. The voltage shift after the initial lithiation was mainly caused by the structure and stress change in the first conversion process [46]. The reversible conversion between FeS and Fe enabled FeS@C electrode to possess great reversibility of the lithium insertion/extraction behavior, which was further confirmed by nearly overlapping CV curves in the 2nd–5th cycles. The CV curves of the FeS@C-600 and FeS@C-800 electrodes (Figure S11) also show similar curves to the FeS@C-700 electrode, indicating good electrochemical reversibility of Li storage for all of these electrodes.

The electrochemical performances of these FeS@C electrodes were evaluated by galvanostatic charge/discharge measurements. Figure 4b shows the first charge/discharge curves of FeS@C electrodes at a current density of 100 mA g^−1^. The FeS@C-600, FeS@C-700, and FeS@C-800 electrodes displayed high reversible capacities of 1497, 1478, and 1239 mAh g^−1^ with an initial coulombic efficient (ICE) of 52.38%, 56.74%, and 50.44%, respectively. The specific capacity of all FeS@C electrodes was calculated by the mass of FeS@C particles. The FeS@C electrodes possessed low initial coulombic efficiency, which may be caused by formation of the solid–electrolyte interface (SEI) and the irreversible reaction between Li and FeS [29], which was confirmed by the CV curves of the FeS@C particles (Figure 4a and Figure S11, Supporting Information). The coulombic efficient of these electrodes quickly exceeded 90% in the second cycle and 96%–99.86% in the later cycles (Figure S12, Supporting Information), indicating a highly stable electrochemical performance.

In addition, FeS@C electrodes also showed good cycling performance. Figure 4c presents the cycle performance of FeS@C-600, FeS@C-700, and FeS@C-800 electrodes at a current density of 100 mA g^−1^. We observe that the FeS@C-700 electrode delivered an initial discharge capacity of 1478 mAh g^−1^ at a current density of 100 mA g^−1^. The capacity faded in the first 10 discharge–charge cycles, and was gradually stable in the later cycles. After 100 charge–discharge cycles, the FeS@C-700 electrode held a reversible discharge capacity of 800 mAh g^−1^, which is higher than that of the FeS@C-600 (655 mAh g^−1^) and FeS@C-800 electrodes (547 mAh g^−1^). The relatively long-term cycle stability of these FeS@C electrodes was also evaluated at a high current density of 500 mA g^−1^ for 500 cycles. The FeS@C-700 electrode delivered a high specific capacity of 640 mAh g^−1^ with a capacity retention of 100% (compared with the 2nd discharge capacity) at a high current density of 500 mA g^−1^ after 500 cycles. As can be seen in Figure 4e and Figure S13 (Supporting Information), these three electrodes showed a stable cycle performance even at high current density. It should be pointed out that the Super P carbon black used in these electrodes also has a capacity contribution [47]. SEM images of the FeS@C-700 electrode before and after 500 cycles at the current density of 500 mA g^−1^ are shown in Figure S14 (Supporting Information). The complete electrode can be observed in Figure S14b (Supporting Information). These FeS@C electrodes exhibited a good cycle performance, which may be ascribed to their hierarchical structure with large surface area and high pore volume. The porous carbon matrix provided a highly conductive network for rapid electron transfer and shortened ion diffusion pathway for fast ion transport. More importantly, FeS nanoparticles nanoconfined in the porous carbon matrix can not only buffer the large volume expansion/shrinkage of FeS particles but can also reduce polysulfide dissolution into the electrolyte. The unique properties of the FeS@C particles enable a remarkable rate capability and excellent cycling performance.

## 4. Conclusions

In summary, we have developed a simple, low-cost, and efficient approach to prepare the FeS@C particles by direct pyrolysis of iron(III) p-toluenesulfonate hexahydrate. The FeS nanoparticles are nanoconfined into hierarchical amorphous carbon during the pyrolysis process. Owing to their hierarchical structure, the resultant FeS@C particles show high specific capacity, good rate performance, and long-term stability as lithium-ion battery anodes. This work represents a major breakthrough for the mass production of low-cost anode materials for high-density lithium-ion batteries.

## Supplementary Materials

The following are available online at https://www.mdpi.com/2079-4991/9/10/1467/s1, Figure S1: Digital photo images of (a) iron (III) p-toluenesulfonate hexahydrate (IPTH) precursor and (b) FeS@C particles from IPTH annealed at 700 °C, Figure S2: TG and DTG curves of iron(III) p-toluenesulfonate hexahydrate under Ar atmosphere with a ramp rate of 5 °C min^-1^, Figure S3: Digital photo images of FeS@C particles before (a) and after (b) pyrolysis at 700 °C, Figure S4: SEM images of FeS@C-700 particles at increasing magnification, Figure S5: Raman spectrum of FeS@C-700 particles, Figure S6: (a) Nitrogen adsorption/desorption isotherms and (b) corresponding pore size distribution for FeS@C-600 and FeS@C-800 particles, Figure S7: Survey XPS spectra of the FeS@C-600, FeS@C-700 and FeS@C-800 particles, Figure S8: High-resolution XPS spectra of Fe 2p, S 2p and C 1s for FeS@C-600 (a, c, e) and FeS@C-800 (b, d, f) particles, Figure S9: Electrochemical impedance spectra (EIS) of the FeS@C-600, FeS@C-700 and FeS@C-800 electrode before cycling, Figure S10: TGA curves of FeS@C-600, FeS@C-700 and FeS@C-800 particles under O_2_ atmosphere with a ramp rate of 10 °C min^-1^, Figure S11: Representative cyclic voltammograms curves of (a) FeS@C-600 and (b) FeS@C-800 electrodes at a scan rate of 0.02 mV s^-1^, Figure S12: Coulombic efficiency of FeS@C-600, FeS@C-700 and FeS@C-800 electrodes, Figure S13: Charge/discharge profiles of (a) FeS@C-600 and (b) FeS@C-800 electrodes at a current density of 500 mA g^-1^, Figure S14: SEM images of the FeS@C-700 electrode before (a) and after (b) 500 cycles at a current density of 500 mA g^-1^ over the potential window of 0.01– 3 V (versus Li/Li+).
