# Supplementary material for: Facile Synthesis of FeS@C Particles Toward High-Performance Anodes for Lithium-Ion Batteries
Source: Nanomaterials (Basel). 2019 Oct 16;9(10):1467. doi: 10.3390/nano9101467 (PMC6835662; doi:10.3390/nano9101467)
Supplement: Supplementary file 1 [file nanomaterials-09-01467-s001.pdf]

nanomaterials

Supporting Information

Facile Synthesis of FeS@C Particles Toward High-Performance Anodes for Lithium-Ion Batteries

Xuanni Lin [1], Zhuoyi Yang [1], Anru Guo [2,*] and Dong Liu [1,*]

[1] College of Chemical Engineering, Beijing University of Chemical Technology, Beijing 100029, China; Elainelxn@126.com (X.L.); yegbert@163.com (Z.Y.)
[2] Aerospace Research Institute of Materials & Processing Technology, Beijing 100076, China
* Correspondence: 200521025@163.com (A.G.); liudong@mail.buct.edu.cn (D.L.); Tel.: +86-010-68380659 (A.G.); +86-010-64437921 (D.L.)

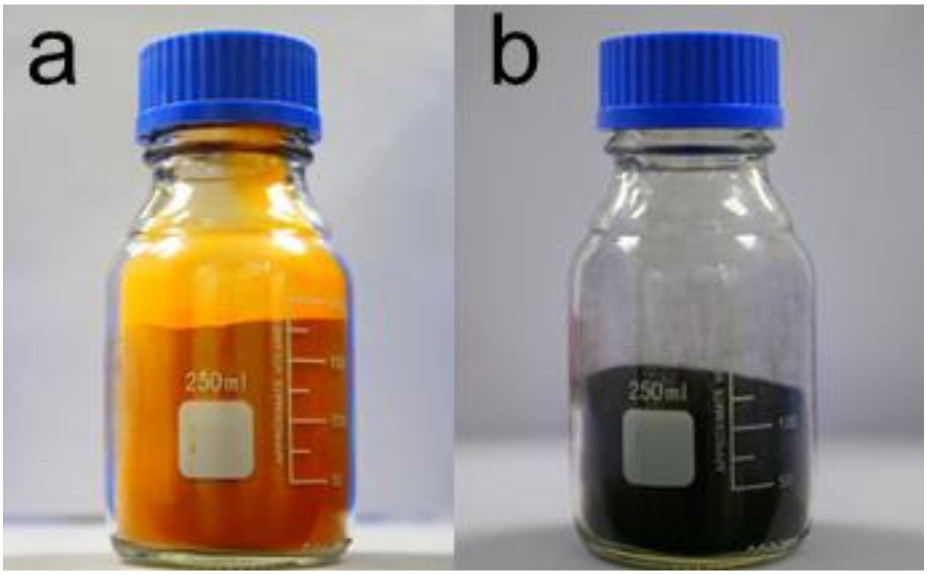

Figure S1. Digital photo images of (a) iron (III) p-toluenesulfonate hexahydrate (IPTH) precursor and (b) FeS@C particles from IPTH annealed at 700 °C. The yield of the FeS@C particles at 700 °C is 52%.

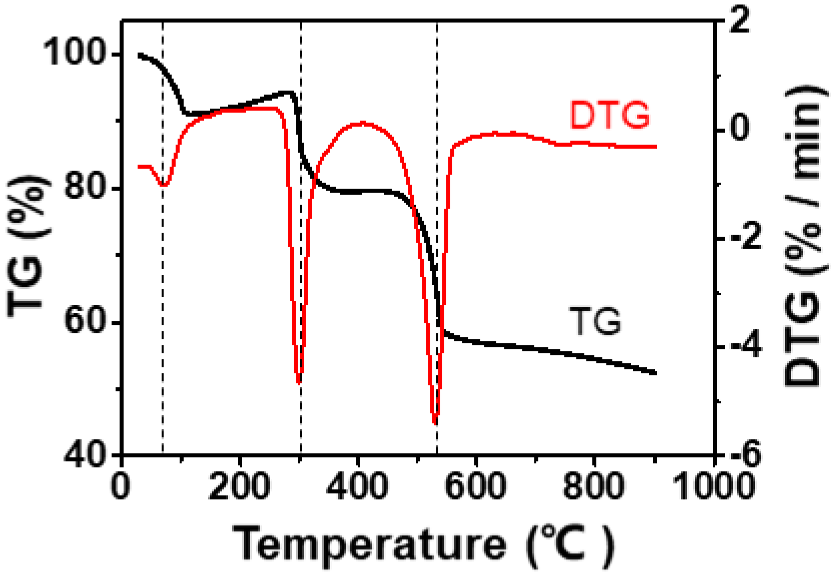

Figure S2. TG and DTG curves of iron(III) p-toluenesulfonate hexahydrate under Ar atmosphere with a ramp rate of 5 °C min^{-1}.

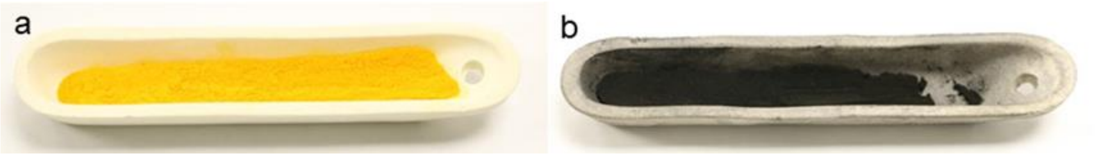

Figure S3. Digital photo images of FeS@C particles before (a) and after (b) pyrolysis at 700 °C.

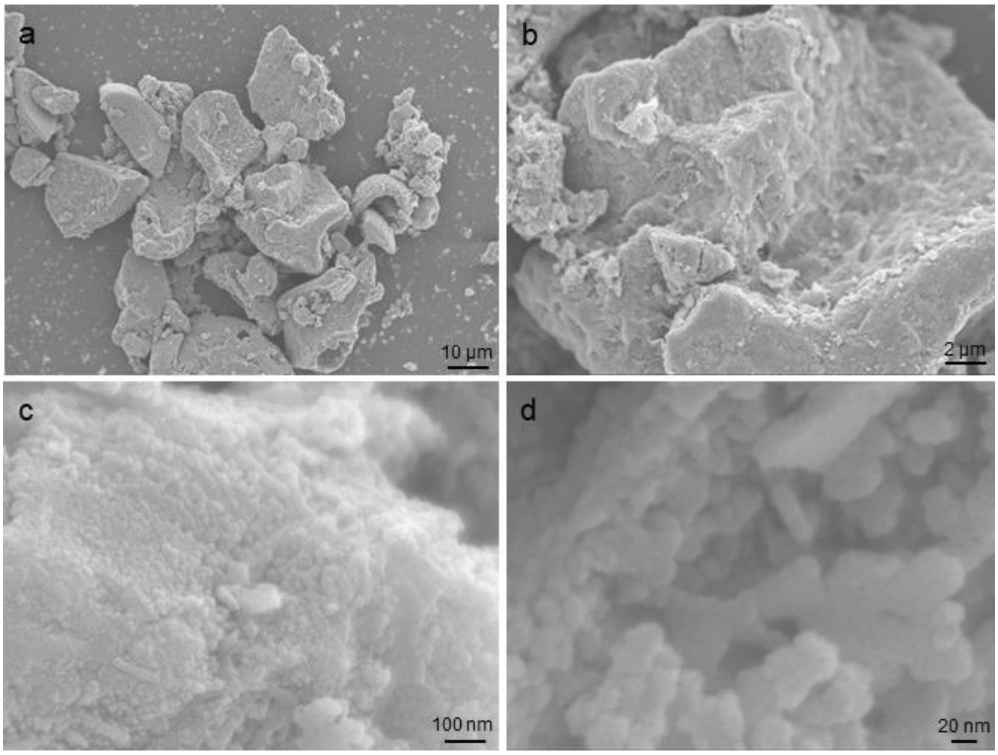

Figure S4. SEM images of FeS@C-700 particles at increasing magnification.

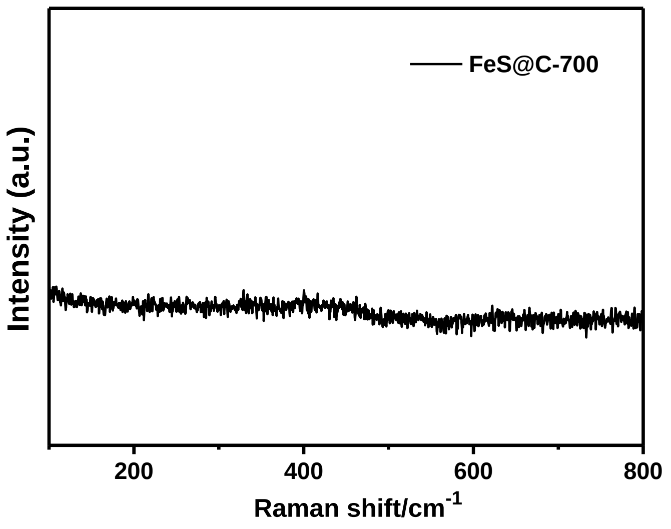

Figure S5. Raman spectrum of FeS@C-700 particles.

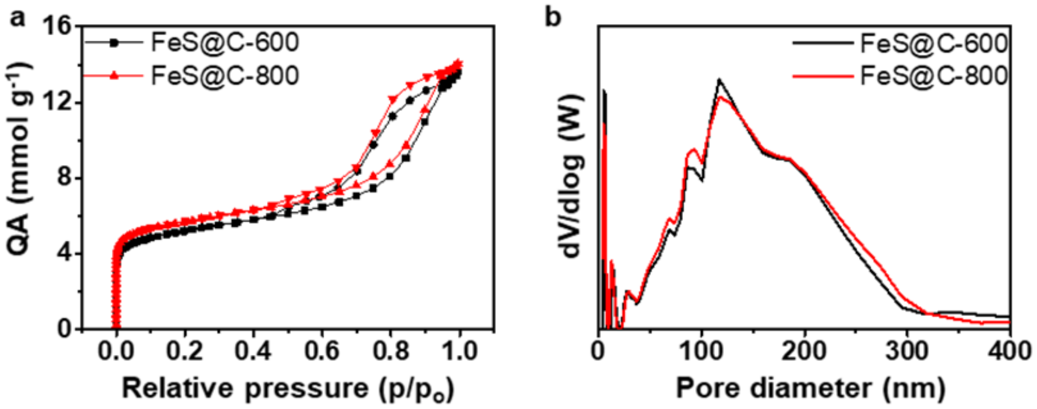

Figure S6. (a) Nitrogen adsorption/desorption isotherms and (b) corresponding pore size distribution for FeS@C-600 and FeS@C-800 particles.

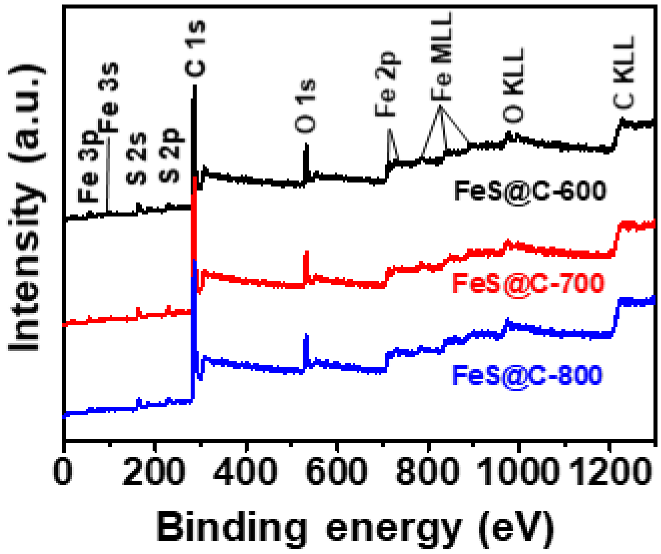

Figure S7. Survey XPS spectra of the FeS@C-600, FeS@C-700 and FeS@C-800 particles.

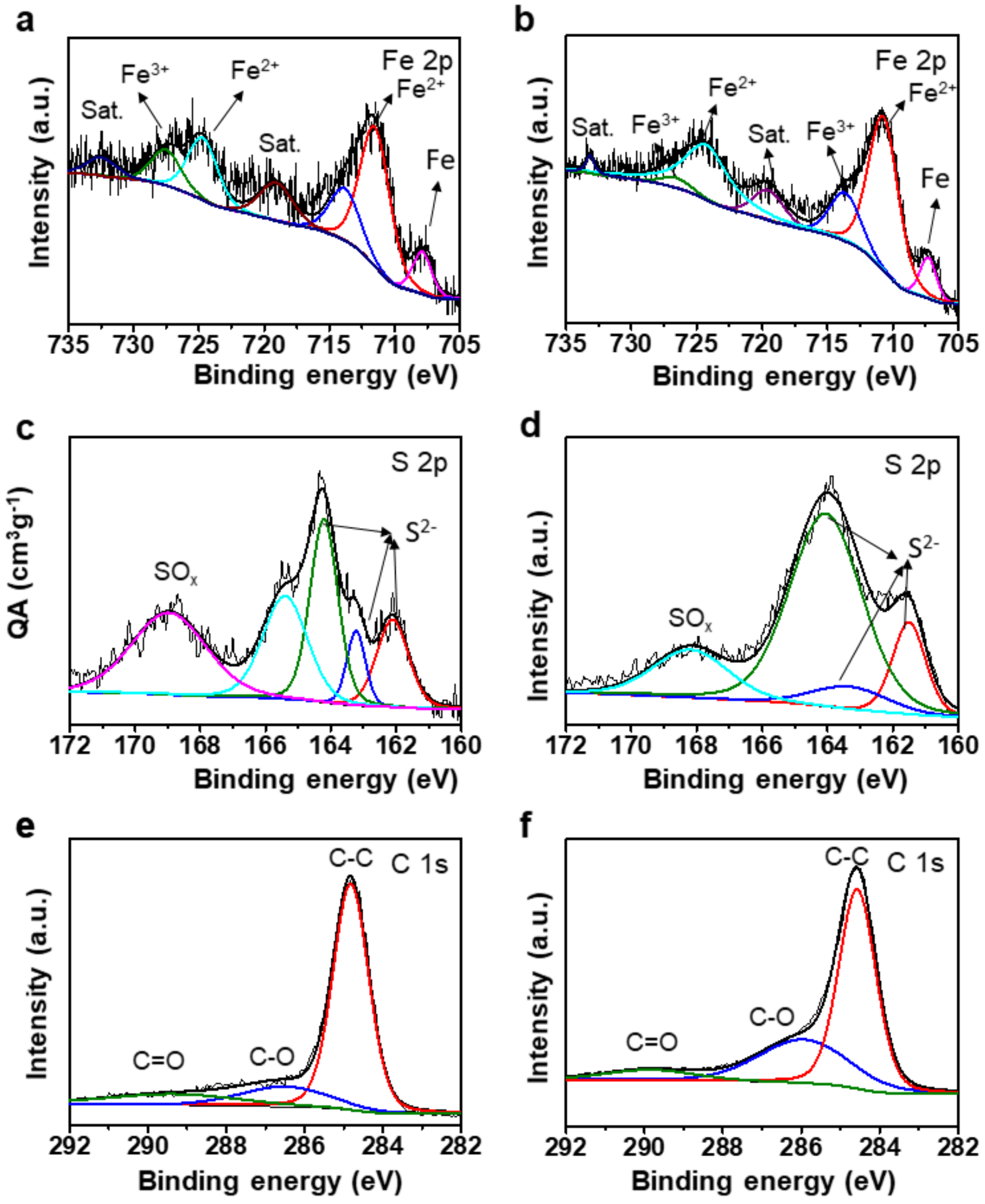

Figure S8. High-resolution XPS spectra of Fe 2p, S 2p and C 1s for FeS@C-600 (a, c, e) and FeS@C-800 (b, d, f) particles.

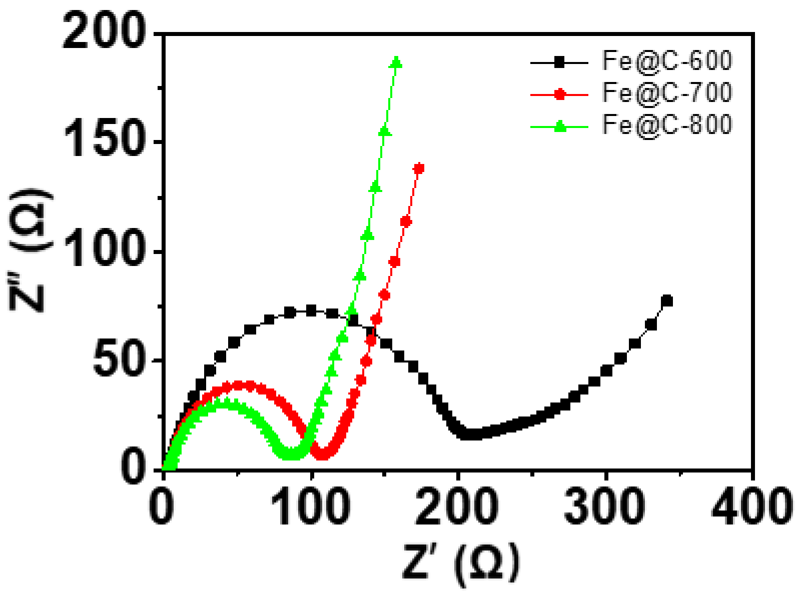

Figure S9. Electrochemical impedance spectra (EIS) of the FeS@C-600, FeS@C-700 and FeS@C-800 electrode before cycling.

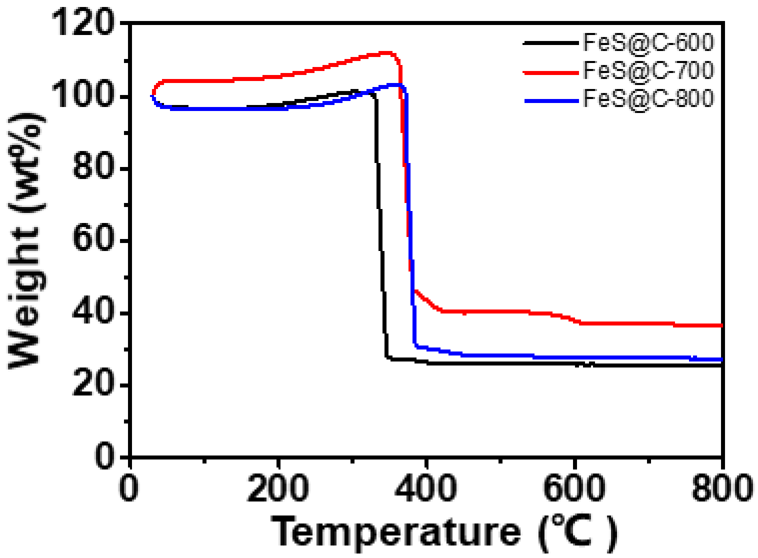

Figure S10. TGA curves of FeS@C-600, FeS@C-700 and FeS@C-800 particles under O_2 atmosphere with a ramp rate of 10 °C min^{-1}.

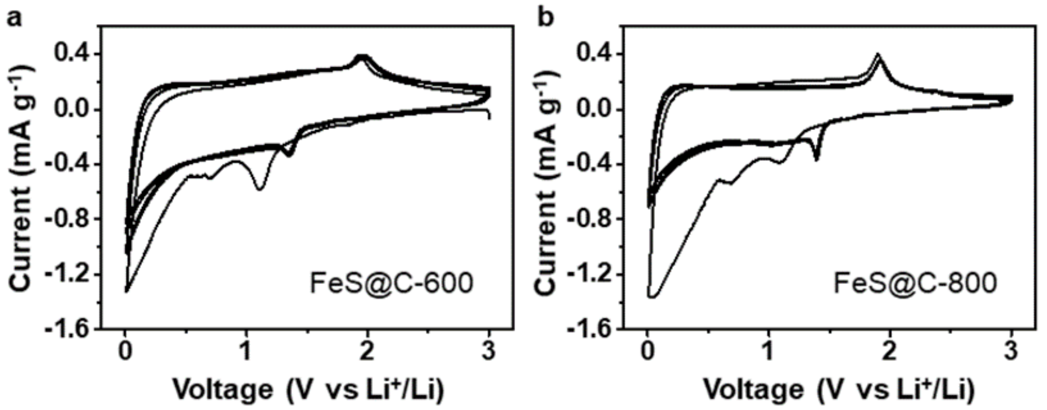

Figure S11. Representative cyclic voltammograms curves of (a) FeS@C-600 and (b) FeS@C-800 electrodes at a scan rate of 0.02 mV s^{-1}.

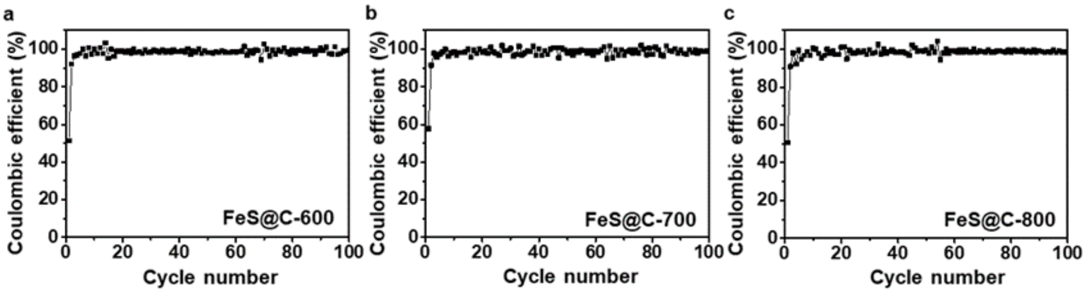

Figure S12. Coulombic efficient of FeS@C-600, FeS@C-700 and FeS@C-800 electrodes.

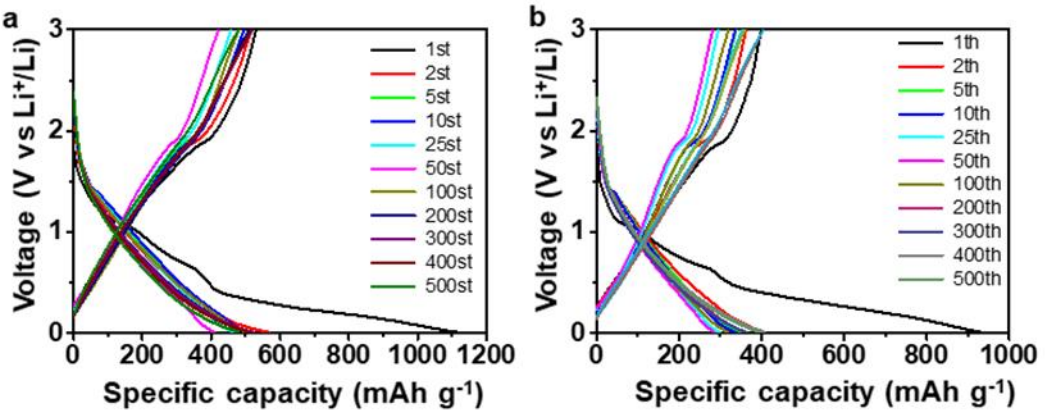

Figure S13. Charge/discharge profiles of (a) FeS@C-600 and (b) FeS@C-800 electrodes at a current density of 500 mA g^{-1}.

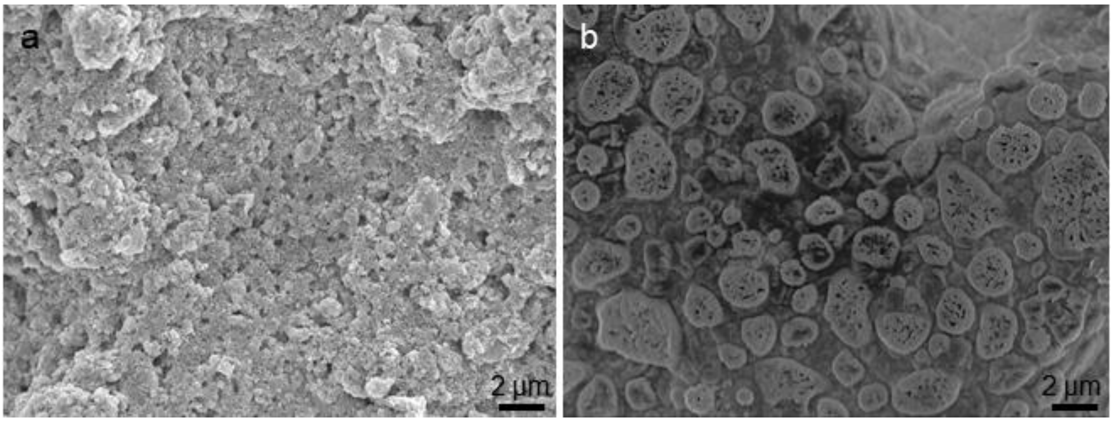

Figure S14. SEM images of the FeS@C-700 electrode before (a) and after (b) 500 cycles at a current density of 500 mA g^{-1} over the potential window of 0.01– 3 V (versus Li/Li^{+}).
